# Ubiquitin-Like Modifiers: Emerging Regulators of Protozoan Parasites

**DOI:** 10.3390/biom10101403

**Published:** 2020-10-03

**Authors:** Maryia Karpiyevich, Katerina Artavanis-Tsakonas

**Affiliations:** Department of Pathology, University of Cambridge, Cambridge CB2 1QP, UK

**Keywords:** ubiquitin-like, SUMO, NEDD8, ATG8, ATG12, URM1, UFM1, parasite, protozoa, post- translational regulation

## Abstract

Post-translational protein regulation allows for fine-tuning of cellular functions and involves a wide range of modifications, including ubiquitin and ubiquitin-like modifiers (Ubls). The dynamic balance of Ubl conjugation and removal shapes the fates of target substrates, in turn modulating various cellular processes. The mechanistic aspects of Ubl pathways and their biological roles have been largely established in yeast, plants, and mammalian cells. However, these modifiers may be utilised differently in highly specialised and divergent organisms, such as parasitic protozoa. In this review, we explore how these parasites employ Ubls, in particular SUMO, NEDD8, ATG8, ATG12, URM1, and UFM1, to regulate their unconventional cellular physiology. We discuss emerging data that provide evidence of Ubl-mediated regulation of unique parasite-specific processes, as well as the distinctive features of Ubl pathways in parasitic protozoa. We also highlight the potential to leverage these essential regulators and their cognate enzymatic machinery for development of therapeutics to protect against the diseases caused by protozoan parasites.

## 1. Introduction: When Parasitic Protozoa Met Ubls

**Figure 1:**
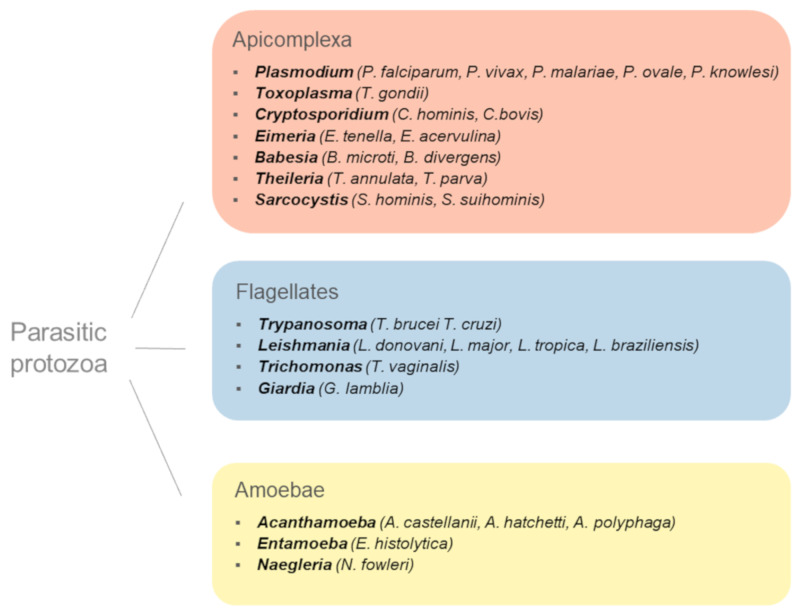
Parasitic protozoa of medical and veterinary importance.

Parasitic protozoa are a diverse polyphyletic group of unicellular eukaryotes that have adapted to live in the cells, tissues, or organs of host organisms [1]. These parasites cause diseases of medical and veterinary importance and impose a significant socioeconomic burden worldwide (Figure 1). Five species of *Plasmodium* genus are the etiological agents of malaria, a devastating disease that affects over 200 million people and claims the lives of over 400 thousand people annually [2]. Human African trypanosomiasis, Chagas disease, and leishmaniasis, caused by *Trypanosoma brucei*, *Trypanosoma cruzi*, and *Leishmania* spp., respectively, are classed as neglected tropical diseases and lead to considerable morbidity and mortality in developing countries [3,4,5]. A common parasitic zoonosis is associated with *Toxoplasma gondii*, a protozoan parasite that is capable of infecting warm-blooded animals and humans and may cause congenital disease, abortion, or severe complications in immunocompromised individuals [6]. Enteric protozoans *Cryptosporidium* spp., *Giardia lamblia*, and *Entamoeba histolytica* cause diarrhoeal illnesses in humans, as well as companion and farm animals. While mainly manifesting as self-limiting diarrhoea, these infections may become disabling or fatal for very young or immunocompromised individuals and lead to severe complications, such as amoebic brain abscesses and necrotising colitis in *E. histolytica*-infected patients [7]. Trichomoniasis, one of the most common sexually transmitted diseases, is caused by *Trichomonas vaginalis*, a protozoan parasite that remains understudied despite being globally widespread and linked to adverse pregnancy outcomes and increased HIV susceptibility [8]. *Naegleria fowleri*, the causative agent of a rare, but highly lethal, primary amoebic meningoencephalitis (PAM) with a mortality rate estimated at 95–97%, may be considered an emerging pathogen as there are indications of an upward trend in the number of PAM cases over the last two decades and an increasing favourability of environmental conditions for the growth of this parasite [9]. Lack of vaccines, limited chemotherapy options, and the emergence of drug-resistant parasites are the challenges posed by the majority of protozoan diseases. There is a clear clinical need for the development of novel drugs targeting parasitic protozoa in order to prevent and treat these diseases [10,11].

Many protozoan parasites have intricate life cycles involving progression through multiple morphologically and physiologically distinct stages, which requires complex regulation of their gene expression. Notably, kinetoplastid parasites, such as *Leishmania* and *Trypanosoma*, lack regulatory transcription factors and rely predominantly on polycistronic transcription, resulting in a severely limited ability to exert transcriptional regulation [12,13]. Furthermore, in *Plasmodium*, a considerable discrepancy between the abundance of mRNAs and corresponding proteins has been demonstrated, with the protein abundance peaking on average 11 hours after the peak of the corresponding transcript [14]. These observations indicate that the regulation of gene expression in protozoan parasites does not solely rely on transcriptional control mechanisms. Instead, the dynamics of translation, protein degradation, and post-translational modifications (PTMs) are likely to be key regulators of protein expression at the relevant life cycle stages. A wide range of PTMs have been reported in protozoans, including phosphorylation, acetylation, succinylation, palmitoylation, crotonylation, malonylation, methylation, glycosylation, ubiquitination, and ubiquitin-like (Ubl) modifications [15,16,17]. 

‘Ubl’ is a term that encompasses protein modifiers which are structurally and evolutionarily related to ubiquitin. The Ubl family includes neural precursor cell-expressed developmentally downregulated 8 (NEDD8), small ubiquitin-related modifier (SUMO), ubiquitin-fold modifier 1 (UFM1), ubiquitin-related modifier 1 (URM1), autophagy-related proteins 8 and 12 (ATG8 and ATG12), interferon-stimulated gene 15 (ISG15), and human leukocyte antigen-F adjacent transcript 10 (FAT10) [18]. A rich variety of Ubls have been found in parasitic protozoa [19,20,21,22], with the exception of ISG15 and FAT10 (Table 1). These two modifiers have been implicated in immune and stress responses in the context of multicellular organisms [23,24], which may explain their absence in unicellular parasitic protozoans.

Ubls share certain notable features with ubiquitin, namely the characteristic five-stranded β-grasp fold [25] and requirement for a mechanistically conserved enzymatic cascade to mediate their attachment to substrates. Ubiquitin and the majority of Ubls are covalently attached to their targets, mainly lysine residues of protein substrates, via a three-step process sequentially catalysed by the modifier-specific E1 activating, E2 conjugating, and E3 ligase enzymes. The formation of polymeric chains, resulting from additional moieties being conjugated onto the internal acceptor sites of the modifier itself, has been described for ubiquitin and some Ubls, such as SUMO, NEDD8, and UFM1 [26]. The dynamic nature of ubiquitin and Ubl modifications is underpinned by the existence of deubiquitinases (DUBs) and Ubl-specific proteases (Ulps) that can remove the respective modifications from their substrates. Additionally, some DUBs and Ulps are responsible for cleaving the C-termini of ubiquitin and Ubl precursors to release mature forms of these modifiers, containing a C-terminal glycine (Gly) or diglycine (Gly-Gly) motif that is required for activation and conjugation to substrates [27]. The translation of URM1, ATG12, and FAT10 mRNAs produces mature forms that do not undergo proteolytic processing [26]. 

Ubl modifications allow the fine-tuning of their targets by altering their stability, conformation, intracellular compartmentalisation, or affinity to binding partners. Consequently, by changing the features of their substrates, Ubls regulate numerous cellular processes, including cell cycle progression, transcription, stress responses, DNA damage repair, cell signalling, nuclear transport, and autophagy [18,28].

While robust mass spectrometry-based proteomic techniques have been devised to detect and quantify certain PTMs, such as phosphorylation, other types of modifications, notably Ubls, remain a challenge to measure directly [29,30]. There has been great progress in developing methods for global analysis of ubiquitinated proteins [30], and multiple ubiquitome datasets were released for various organisms, including parasitic protozoa [31,32]. However, detection of endogenous Ubl-modified targets is a more complicated task due to the low abundance and transient nature of these modifications. This issue can be mitigated by using sophisticated sample enrichment and processing methods, many of which rely on the availability of highly specific reagents (e.g., monoclonal SUMO antibodies) [33]. Additionally, the shared Gly-Gly C-terminal remnant produced upon trypsin digestion of modified substrates in the course of sample preparation for mass spectrometry of NEDD8 and ISG15-modified proteins, as well as ubiquitin-modified ones, further stunts investigations. The Gly-Gly signature is a useful feature for the detection of modified peptides in mass spectrometry and is widely used for assigning ubiquitination sites [29,30]. As a result, NEDD8 and ISG15 modifications may be overlooked, obscured by more abundant ubiquitin ones, and require complex interventions to ascertain, such as the use of modified NEDD8 enzymatic machinery [34] and NEDDylation inhibitors [35].

Where experimental data are lacking, the predictive power of bioinformatics can be harnessed to guide experimental research. A variety of models for predicting PTM sites on protein substrates have been developed [36], some of which were applicable to parasitic protozoa. In particular, algorithms based on amino acid sequence motifs [36,37], protein secondary structure [38,39], or a combination of both [40], have been devised to determine SUMOylation sites. Enabled by the release of protozoan proteomic and genomic datasets over the last two decades [41,42,43], computational approaches have been instrumental in identifying putative Ubls and enzymes mediating Ubl cascades in these organisms based on sequence and structural similarity to relevant conserved domains [19,20,21,22]. However, due to significant divergence from model organisms and a high proportion of proteins without any known homology, functional studies are critical to elucidating these pathways in parasitic protozoa.

Research into the functional aspects of ubiquitin and Ubl pathways has been accelerated by the development of activity-based probes [44,45,46] and inhibitors [47,48,49,50] that target modifier-specific enzymatic machinery with high specificity. These chemical tools, often coupled with mass spectrometry and X-ray crystallography, enable the capture, identification, and structural and functional characterisation of enzymes mediating the Ubl cascades. A number of functional studies have successfully used activity-based probes and inhibitors to investigate Ubl pathways in parasitic protozoa [51,52,53,54,55].

Many experimental and computational techniques developed in model organisms are difficult to implement in parasitic protozoa due to striking deviations in their genomes, cellular processes, and regulatory mechanisms. Another obstacle is the complexity of life cycles and environmental requirements of protozoan parasites, making many unsuitable for large-scale cultivation and recapitulation of the complete life cycle in the laboratory. This translates into substantial difficulties in generating sufficient amounts of parasite material for standard workflows. Although protozoan parasites present significant challenges as experimental objects, recently developed genetic approaches, such as CRISPR/Cas genome editing [56] and high-throughput transposon insertional mutagenesis [57], have expanded the possibilities to conduct functional studies in these organisms. 

Although the body of research addressing the complexity of Ubl functions and pathways is steadily growing, it is important to point out that most of the studies are performed using mammalian cells or yeast. Such studies are very powerful in elucidating the details of conserved eukaryotic processes, but may not fully apply to highly divergent and specialised organisms, parasitic protozoa being a prime example. Although underexplored in parasitic protozoa, emerging data indicate that Ubls are involved in novel parasite-specific roles, in addition to fulfilling the conserved “core” functions. Since the ubiquitin pathway in protozoans has been reviewed earlier [58], here we will focus on investigations addressing the more conventional as well as the unexpected functions of Ubls in parasitic protozoa. We will also highlight the distinguishing features of Ubl enzymatic machinery in these unusual organisms and the potential for Ubl pathways to be targeted by current and novel drugs against the diseases caused by parasitic protozoa.

## 2. SUMO: Wrestling with Stress and More

SUMO is one of the most comprehensively studied Ubls that has been found in all eukaryotes to date. Unicellular eukaryotes, such as yeast and protozoa, possess a single SUMO-encoding gene, while vertebrates and higher plants have multiple SUMO paralogs. Attachment of SUMO to lysine residues of protein substrates (SUMOylation) is mediated by the heterodimeric SUMO-activating E1 enzyme that consists of the Uba2 catalytic subunit (i.e., SAE2) and the Aos1 subunit (i.e., SAE1), the SUMO-conjugating E2 enzyme Ubc9, and several SUMO E3 ligases. SUMOylation can occur in an E3-independent manner, provided the Ubc9 concentration is high and the substrate contains a consensus motif ψKxD/E (ψ—a large hydrophobic amino acid; x—any amino acid). Removal of SUMO from modified substrates (deSUMOylation) and maturation of SUMO precursors are mediated by SUMO-specific Ulps, most belonging to sentrin-specific protease (SENP) and permuted papain fold peptidases of dsRNA viruses and eukaryotes (PPPDE) families [59,60]. 

A wide range of proteins have been identified as targets of SUMOylation, linking this PTM to the majority of essential processes in eukaryotic cells. The regulatory role of SUMO has been most extensively studied in nuclear functions, namely transcription, DNA damage repair, chromatin organisation, nuclear transport, and mitosis [60]. It is also becoming evident that there are complex relationships between SUMOylation and other PTMs, in particular phosphorylation, acetylation, and ubiquitination, which expands the scope of SUMO-mediated regulation [59,60]. In many organisms, changes in SUMOylation have been observed in response to oxidative, osmotic, hypoxic, heat, and genotoxic stresses, indicating that SUMO might play a protective role in various types of cellular stress [61]. 

Considerable variation in protein SUMOylation levels in different *P. falciparum* intraerythrocytic stages has been demonstrated. The peak in the amount of high molecular weight *P. falciparum* SUMO (*PfS*UMO) conjugates coincides with the trophozoite stage of the intraerythrocytic development cycle [62]. During this stage, malaria parasites consume large quantities of host haemoglobin and are subject to oxidative stress induced by haemoglobin digestion by-products. Furthermore, *Pf*SUMO conjugation can be increased in a dose-dependent manner upon treatment of parasites with artemisinin, an antimalarial drug that triggers oxidative stress [63]. Therefore, similar to this Ubl in stress responses of other organisms [61], it is likely that increased SUMOylation is a protective response against oxidative stress in *P. falciparum* trophozoites. Notably, downregulation of *Pf*SUMO was reported in *P. falciparum* trophozoites treated with an isocryptolepine derivative ICL-M [64], and may interfere with their ability to mount an efficient stress response, thereby contributing to the lethal effect of the drug.

Indications of a parasite-specific role for SUMO were demonstrated by a proteomic study where 23 proteins emerged as putative *Pf*SUMO substrates in *P. falciparum* [65]. While many matched orthologs of proteins known to be SUMOylated in other organisms, a few comprised novel, parasite-specific targets. Of particular interest is *Pf*Sir2, a nuclear protein involved in transcriptional regulation of *var* genes that confer antigenic variation, implicating SUMOylation in immune evasion. Immunofluorescence staining using a monoclonal antibody against *Pf*SUMO revealed the presence of this Ubl within parasite nuclei and cytoplasm [62]. Interestingly, a separate study using polyclonal antibodies also localised *Pf*SUMO within Maurer’s clefts [65]. These parasite-derived structures are disc-shaped cisterns bound by a single membrane that function as sorting and trafficking hubs for parasitic proteins destined for export into host red blood cells, in particular onto the red blood cell surface, as seen with the *var* gene product, PfEMP1 [66]. It is possible that SUMOylated proteins of parasitic origin are trafficked into erythrocytes. Alternatively, *Pf*SUMO may play a regulatory role in the formation and maintenance of Maurer’s clefts or protein sorting and transport in these structures [65]. 

In silico identification of genes encoding SUMO and the enzymatic machinery required for attachment and removal of this Ubl in *P. falciparum* [19,20,62,65] prompted further research into structural and functional aspects of the SUMO pathway in malaria parasites. In vitro studies of *Pf*SUMO conjugation pathway demonstrated that *Pf*SUMO E1 (heterodimer *Pf*Aos1-*Pf*Uba2) and *Pf*SUMO E2 (*Pf*Ubc9) are catalytically active and can mediate SUMOylation of mammalian RanGAP1, a well-characterised SUMO substrate bearing the ψKxD/E consensus motif. *Pf*SUMO E1 is able to activate both the *P. falciparum* SUMO and the human SUMO-2, and RanGAP1 can be modified with either of them [62,63]. Although the functional interchangeability underscores the evolutionary conservation of this Ubl, the interactions between SUMO E1 and SUMO E2 are organism-specific and determined by divergent E1–E2 binding interfaces in *P. falciparum* enzymes and their human counterparts [62,63]. The presence of multiple amino acid residues on *Pf*Uba2 and *Pf*Ubc9 that dictate the selectivity of their interaction indicates potential for development of parasite-specific inhibitors targeting this pathway [62,63]. Furthermore, mutations in both *Pf*Uba2 and the *Pf*SUMO E3 ligase PIAS arose during selection for resistance to high concentrations of the benzoxaborole drug AN13762, underscoring the pertinence of the entire SUMO pathway to the mode of action of this drug and potentially to other antimalarials [67]. Considering that *Pf*SUMO and the associated enzymatic machinery are essential for survival of *P. falciparum* [57], selective disruption of the key steps in the attachment of this Ubl to substrates is likely to be lethal to malaria parasites. 

In terms of SUMO removal from target proteins, bioinformatics analyses predict two *P. falciparum* SUMO-specific Ulps, *Pf*SENP1 (functionally characterised) and *Pf*SENP2 (uncharacterised) [55]. It was demonstrated that *Pf*SENP1 removes SUMO moieties from modified RanGAP1 and processes SUMO precursors in vitro [55]. Upregulation of this Ulp was observed during the parasite’s intrahepatic stage following disruption by γ-irradiation of *P. falciparum* sporozoites, further supporting a role of SUMOylation in stress response [68]. Highlighting this enzyme’s potential as a drug target, the substrate sequence specificity of *Pf*SENP1 was shown to differ significantly enough from that of human SENPs, that a class of aza-epoxide small molecule inhibitors were able to specifically block replication of *P. falciparum* in erythrocytes, with parasite clearance and potency of *Pf*SENP1 inhibition showing a direct correlation [55]. Although *Pf*SENP2 has not been tested in vitro, the H423Y variant of this enzyme was identified by retrospective longitudinal genome surveillance as a candidate background mutation contributing to the spread of Kelch13-mediated artemisinin resistance in *P. falciparum* [69]. Assuming *Pf*SENP2 is confirmed as a deSUMOylase, this finding suggests that the SUMO pathway is involved in boosting the fitness of artemisinin-resistant parasite strains. Moreover, *Pf*SENP2 has a potential to serve as a molecular marker for drug resistance surveillance, facilitating the efforts to trace and mitigate the spread of artemisinin resistance in *P. falciparum* populations [69].

A comprehensive study of a SENP family protease of *T. gondii*, *Tg*Ulp1, revealed that this enzyme is catalytically active, and its expression is intricately regulated by two non-coding RNAs: *Tg*-miR-60 and *TgUlp1*-NAT1 [70]. *Tg*-miR-60, the most abundant microRNA (miRNA) species in *T. gondii*, negatively regulates *Tg*Ulp1 expression and is the first *T. gondii* miRNA to be assigned a target. *TgUlp1*-NAT1 is a polyadenylated antisense RNA that is also likely to act as a negative regulator of *Tg*Ulp1 expression [70]. The existence of such sophisticated mechanisms to modulate the expression of *Tg*Ulp1 suggests that *T. gondii* SUMOylation is a highly complex process with multiple layers of regulation and clearly deserves further investigation. 

While only 23 proteins emerged as putative *Pf*SUMO substrates in *P. falciparum* [65], a similar approach identified 120 candidate substrates in *T. gondii*, many of which were consistent with SUMOylated orthologs in other organisms [71]. The timing of SUMOylation in *T. gondii* is tightly controlled and responsive to specific cues, exemplified by the transient and considerable increase in *Tg*SUMO conjugates at the parasite surface during host cell invasion [71]. Furthermore, *T. gondii* subjected to pH stress to induce in vitro conversion from replicative tachyzoite to latent (cyst) bradyzoite stage accumulated *Tg*SUMO conjugates at the bradyzoite parasitophorous vacuole membrane (PVM). Additionally, SUMOylation was observed at the wall of tissue cysts formed by *T. gondii* bradyzoites in the murine brain. These findings suggest that SUMO may play a regulatory role in *T. gondii* stress responses, transition between developmental stages, and cyst formation [71]. It was demonstrated that monensin, an ionophore drug commonly used to treat coccidiosis and effective against *T. gondii*, downregulates *Tg*SUMO [72]. Decreased *Tg*SUMO levels in monensin-treated *T. gondii* may compromise the ability of the parasites to counteract stress, in particular oxidative stress, which is reportedly induced by this drug [73].

SUMO has been actively investigated in flagellate protozoans, particularly *T. brucei* and *T. cruzi*. For both species, SUMO and SUMO-specific enzymatic machinery were bioinformatically predicted [74,75,76,77] and functionally validated using reconstituted systems in vitro or in bacteria [76,78,79]. Conservation of key structural features typical of SUMO orthologs from model organisms was shown for *T. brucei* SUMO by means of NMR spectroscopy [80]. Remarkably, the formation of polymeric chains has been reported for SUMO found in *T. brucei* [78,81] and *T. cruzi* [79], in contrast to apicomplexan parasites that possess SUMO devoid of internal SUMOylation motifs and likely lacking a basis for polymeric chain assembly [20]. Although the ability to form poly-*Tb*SUMO chains was shown not to be essential for survival of *T. brucei* in culture, it was implicated in telomere positioning, possibly through modulation of protein interaction platforms that are involved in chromatin organisation [81].

An early mass spectrometry analysis revealed 236 proteins as potential SUMOylation substrates in *T. cruzi* [77]; however, upon applying an improved workflow to the experimental dataset, the number of predicted substrates was reduced to only seven [82]. This dramatic difference in number of predicted substrates exemplifies how application of different analytical methods can change outcomes drastically and underscores the need for further experimental validation of mass spectrometry data. In *T. brucei,* 45 proteins with a total of 53 mapped SUMO acceptor sites were unambiguously identified [83]. Proteins associated with nuclear processes accounted for a high proportion of detected substrates, suggestive of a crucial regulatory function in the nucleus of *T. brucei*.

Indeed, a central role in cell cycle control has been demonstrated across the *T. brucei* life cycle. Silencing of *Tb*SUMO in the proliferative procyclic form (within the midgut of the invertebrate host, the tsetse fly) results in growth inhibition, mitotic arrest at the G2/M phase, and failure to properly segregate the chromosomes, although cytokinesis does still proceed [74]. Additionally, the Aurora-like kinase essential for mitotic spindle assembly, chromosome segregation, and cytokinesis in *T. brucei*, *Tb*AUK1, required SUMOylation to properly function in procyclic parasites [84]. Knockdown of *Tb*SUMO in the bloodstream form (within the vertebrate host) resulted in growth cessation and accumulation of multinucleated cells, indicative of nuclear division in the absence of cytokinesis [85]. Notably, while faithful chromosome segregation in numerous other organisms is dependent on SUMOylation of DNA topoisomerase-II [86,87], the *T. brucei* ortholog of this enzyme showed no evidence of being regulated by *Tb*SUMO. The centromere-localised cleavage activity of *T. brucei* topoisomerase-II that remains intact in the bloodstream form of the parasite depleted of *Tb*SUMO [85] may explain why nuclear division still proceeds in these mutants. Taken together, these data suggest different substrates and functions for SUMO throughout the life cycle of *T. brucei*.

SUMO has also been implicated in antigenic variation of the *T. brucei* bloodstream form through its regulatory role in Variant Surface Glycoprotein (VSG) expression [88]. *T. brucei* antigenic variation relies on the presence of more than a thousand *VSG* genes that display mutually exclusive expression and continuously switch to evade host immune responses [89]. It was demonstrated that a high level of chromatin SUMOylation was associated with the single transcriptionally active *VSG* expression site (*VSG-*ES) in the *T. brucei* nucleus [88]. In contrast, SUMO modification of transcriptional factors and chromatin is more commonly linked to gene repression in other organisms. The enrichment of *Tb*SUMO conjugates in the *T. brucei* nucleus was unique to the active *VSG-*ES and involved the chromatin regions upstream of the *VSG-*ES promoter. *Tb*SIZ1/PIAS1 was identified as the SUMO E3 ligase that mediates the conjugation of *Tb*SUMO onto the chromatin of *VSG-*ES, which leads to its transcriptional activation via RNA polymerase I recruitment. Notably, *Tb*SIZ1-dependent SUMOylation of *Tb*RPA1, the largest subunit of RNA polymerase I, was detected [88], and *Tb*SIZ1 itself appears on the list of reported SUMO substrates [83]. Furthermore, it was demonstrated that *Tb*SUMO-modified nuclear protein SNF2PH is recruited to the active *VSG-*ES promoter to maintain RNA polymerase I, and thus, acts as a transcriptional activator to ensure monoallelic expression of *VSG* [90]. Taken together, these observations suggest that transcriptional activation of *T. brucei VSG* is potentially underpinned by multiple different mechanisms of SUMO-mediated regulation. Considering the limited capacity of trypanosomes to regulate gene expression at the level of transcription, the unusual approach to transcriptional activation of *VSG* highlights the surprising versatility of these biologically unique eukaryotes. SUMO does appear to fulfil more conventional functions within trypanosomes as well, seeing as hydrogen peroxide-induced oxidative stress in *T. brucei* triggered increased SUMOylation, suggestive of a role in managing stress-induced damage [75].

Although SUMO was predominantly enriched in the nucleus of *T. cruzi* [77,79] and *T. brucei* [74,88], *TcSUMO* was also observed to localise to the flagellum, and the pattern of its localisation was dependent on the developmental stage of *T. cruzi* [79]. PFR1, a major protein component of the paraflagellar rod, a lattice-like structure running alongside the axoneme [91], was confirmed as a SUMOylation substrate in *T. cruzi*, which points to the involvement of *Tc*SUMO in flagellar homeostasis and motility [79]. 

The presence of genes encoding SUMO and SUMO-specific enzymatic machinery [21,92], as well as cytoplasmic and nuclear localisation of this Ubl, have been reported for another flagellate protozoan, *G. lamblia*, in the motile trophozoite form [92]. Knockdown of *Gl*SUMO led to alteration of trophozoite cell morphology and distortion of the ventral adhesive disk, a unique and essential structure that enables attachment of the parasite to the intestinal mucosa of the host. Furthermore, *Gl*SUMO-deficient *G. lamblia* trophozoites displayed decreased proliferative ability and cell cycle arrest at G1/S phase, which resembles the effects of SUMO knockdown in trypanosomes [93]. It was also reported that *Gl*SUMO regulates the ability of *G. lamblia* trophozoites to transform into dormant cysts through modification of the parasite’s substrate arginine deiminase (ADI) [94]. SUMOylation of ADI on Lys101 promotes its localisation to the nucleus and leads to downregulation of cyst wall proteins (CWPs) expression, a necessary step for allowing the encystation process to complete. A potential mechanism of ADI-mediated CWP repression is the deimination of histones at the promoters of *cwp* genes, an epigenetic modification that may be conferred through the catalytic activity of ADI in the nucleus [94,95]. Although only 28 proteins were identified as potential substrates by means of mass spectrometry [93], the functional evidence discussed above suggests that *Gl*SUMO is instrumental in many diverse and essential processes to *G. lamblia* development, including cell cycle progression, cell shape control, adhesive disk formation, and encystation.

## 3. NEDD8: Still a Mystery

NEDD8 is the Ubl evolutionarily most closely related to ubiquitin and has largely been studied in the context of its major substrates, Cullins, the scaffold components of the ubiquitin E3 ligase family Cullin-RING E3 ligases (CRLs). Counting over 200 members in mammalian cells, CRLs represent the largest group of RING E3 ligases and control approximately 20% of cellular ubiquitination. Through activation of CRL activity, NEDD8 regulates a wide range of essential processes mediated by CRL substrates, including cell cycle progression, growth and differentiation, stress responses, DNA repair, and cell signalling [96,97]. Although a number of non-Cullin substrates have been reported, such as some E3 ubiquitin ligases, transcription factors, and ribosomal proteins, not all of them are backed by strong experimental evidence due to their use of NEDD8 overexpression [98,99]. The presence of excess NEDD8 can trigger its activation by the ubiquitin E1 enzyme Uba1 and results in erroneous attachment to ubiquitin substrates [98,99]. As such, some of the previously identified non-Cullin substrates require further validation.

NEDD8 is attached to lysine residues of protein substrates through the sequential activity of the heterodimeric NEDD8-activating E1 (NAE) enzyme, composed of the Uba3 catalytic subunit and the Ula1 (also known as NAE1, APPBP1) regulatory subunit, one of two NEDD8-conjugating E2 enzymes (Ubc12/Ube2M or Ube2F), and one of several NEDD8 E3 ligases [98,100]. NEDD8-specific Ulp (deNEDDylase) activity has been demonstrated for two enzymes: NEDP1 (also known as DEN1, SENP8) and COP9 signalosome (CSN). Additionally, a number of DUBs, namely UCHL1, UCHL3, Ataxin 3, and USP21, were reported to cleave NEDD8 [100]. CSN and NEDP1 were proven to be of key functional significance: the former removes NEDD8 from Cullins, while the latter is instrumental in NEDD8 maturation and deNEDDylation of non-Cullin substrates [100]. Despite NEDDylation being an essential process in all eukaryotic organisms (with the exception of *Saccharomyces cerevisiae* [100]), this pathway has not been widely studied in parasitic protozoa thus far.

A notable exception is a study by Liao et al. that functionally characterised NEDD8 in *T. brucei* [101]. The authors demonstrated that although *Tb*NEDD8 can be found throughout the cytosol, it is particularly enriched in the nucleus and flagellum. The significance of this Ubl for nuclear and flagellar processes was further underscored by the appearance of abnormal flagella and mitotic defects in *T. brucei* depleted of *Tb*NEDD8 [101]. Parasites lacking *Tb*NEDD8 displayed flagellar detachment, linking this Ubl to the assembly of the FAZ filament, a cytoskeletal structure that connects the flagellum to the cell body. While disrupted spindle formation and chromosomal segregation are unexpected effects of *Tb*NEDD8 silencing, aberrant re-replication of DNA in mitotic and post-mitotic cells suggests that this Ubl regulates cell cycle progression in *T. brucei,* akin to the role of NEDD8 orthologs in other organisms. NEDDylation was shown to be essential for *T. brucei* survival, and *Tb*NEDD8 knockdown led to a substantial reduction in global protein ubiquitination, highlighting the regulatory role of NEDD8 in the ubiquitin pathway and the close relationship between these PTMs [101]. A total of 70 *Tb*NEDD8-conjugated and *Tb*NEDD8-associated proteins were identified by mass spectrometry using ectopically expressed *Tb*NEDD8, including all six of *T. brucei* Cullins that were further validated as genuine *Tb*NEDD8 substrates [101]. However, due to issues with NEDD8 overexpression, substrates may have been erroneously modified using ubiquitin pathway machinery; therefore, these results should be considered with caution. Liao et al. also analysed affinity-purified proteins from *T. brucei* expressing endogenous levels of tagged *Tb*NEDD8, which revealed 16 proteins, including three Cullins, as putatively *Tb*NEDD8-conjugated or *Tb*NEDD8-associated, and might be a more accurate reflection of the natural substrates [101].

NEDD8-specific enzymatic machinery has also been investigated in *Plasmodium falciparum.* Bioinformatics analyses predicted orthologs of NEDD8, its cognate E1, E2, and E3s [19,20], as well as two Cullins. The enzymes mediating NEDD8 attachment in *P. falciparum* are essential [57], and the parasite is sensitive to the NEDD8 E1 inhibitor MLN4924 [51], indicating that *P. falciparum* requires a functional NEDDylation pathway. Surprisingly, orthologs of key deNEDDylases, NEDP1 and CSN, have not been found in this parasite. Moreover, an in silico analysis failed to identify the CSN subunits in apicomplexan parasites, alluding to either the complete loss of CSN function or the presence of a highly divergent complex in this group of organisms [102]. Remarkably, *P. falciparum* possesses two proteases that are capable of cleaving both ubiquitin and NEDD8 moieties, *Pf*UCHL3 [53,54] and *Pf*UCH37 [52]. While UCHL3 enzymes from other organisms display a dual deNEDDylase/DUB activity, the ability of *Pf*UCH37 to cleave NEDD8 is unusual. In particular, human UCH37 can only cleave ubiquitin, which marks an interesting distinction between the host and the parasite UCH37 enzymes and NEDD8 pathways. The molecular determinants guiding substrate specificity were identified, revealing that a single amino acid residue controls the ability of the enzyme to cleave NEDD8 [51]. Through targeted mutation, it was demonstrated that the deNEDDylase activity of *Pf*UCH37 is dispensable for *P. falciparum* asexual blood stage survival, although its function in other life cycle stages remains to be determined. Since *Pf*UCHL3 is unlikely to cleave NEDD8 from large substrates due to its structural features, deNEDDylation must either transpire via the action of an as of yet unidentified enzyme, or be entirely absent in the asexual stage of parasites. Promisingly, potentially novel deNEDDylases were identified as part of a functional proteomic screen utilising a *P. falciparum*-specific, activity-based probe designed to capture *Pf*NEDD8-reactive enzymes, although their activity remains to be comprehensively validated [51]. 

Curiously, CSN5 (also known as JAB1), the CSN catalytic subunit, was identified in *E. histolytica* as an interacting partner of the parasite-encoded macrophage migration inhibitory factor (MIF), a secreted proinflammatory cytokine implicated in invasion, pathogenesis, and immune evasion [103]. Direct interaction between *Eh*JAB1 and *Eh*MIF blocked the activity of the latter, as demonstrated by the inability of *Eh*MIF to induce IL-8 production by human epithelial cells in the presence of *Eh*JAB1. It was speculated that upon damage of *E. histolytica* cells, normally intracellular *Eh*JAB1 may get released and act to suppress *Eh*MIF-mediated inflammation, thereby creating a negative feedback mechanism. There is also a possibility of *Eh*JAB1 interaction with human MIF, which could have an impact on the host inflammatory and immune responses [103]. 

## 4. ATG8 and ATG12: Moonlighting Autophagy Machinery

ATG8 and ATG12 have been extensively studied, mostly in the context of autophagy, an essential cellular process mediated by these Ubls. Autophagy involves engulfment of cytosolic components, which may include macromolecules, organelles, and pathogens, in double-membrane vesicles, referred to as autophagosomes, followed by fusion with lysosomes and subsequent degradation of the contents. This catabolic process eliminates excessive, defective, or toxic cytosolic components and recycles their degradation products as energy and metabolite sources, thereby maintaining cellular homeostasis and contributing to adaptive responses to stress, such as starvation [104].

ATG8 and ATG12 conjugation systems are functionally related and play a crucial role in autophagosome formation [105]. The covalent attachment of ATG12 to its protein substrate ATG5 is dependent on the E1 enzyme ATG7 and E2 enzyme ATG10, but does not involve an E3 ligase. The ATG12–ATG5 conjugate recruits ATG16L1 to form the ATG12–ATG5–ATG16L1 oligomeric complex that functions as an E3 ligase in the ATG8 conjugation pathway. Unlike most Ubls that modify protein substrates, ATG8 targets the head group of the phospholipid phosphatidylethanolamine (PE), which results in the attachment of ATG8 to the cytosolic face of the autophagosomal membrane—a process that underlies autophagosome formation. The conjugation of ATG8 to PE, also referred to as lipidation, is facilitated by ATG7 (E1), ATG3 (E2), and the aforementioned ATG12-ATG5-ATG16L1 complex (E3), while ATG4 mediates ATG8 precursor processing and removal from substrates (delipidation) [105]. Remarkably, in mammals, both *ATG8* and *ATG4* genes are expanded to include multiple paralogs that display a certain degree of functional specialisation [18].

Bioinformatics analyses revealed that some core autophagy machinery, notably the ATG8 conjugation system, is conserved in most parasitic protozoa [18,106,107]. However, there is ample evidence of substantial loss (particularly in *G. lamblia*), significant divergence (in apicomplexans), or elaboration (expansion of *ATG8* family to include 25 paralogs in *Leishmania* [108]) of key proteins mediating autophagy, indicating that the mechanism and functions of autophagy may be different in these organisms [18,106,107]. Nevertheless, instances of autophagy-like processes, often supported by the presence of ATG8-positive structures, and their functional significance, have been reported for some parasitic protozoa. Examples include the encystation-associated autophagy-like process in *Entamoeba* [109], *Acanthamoeba* [110], and *Naegleria* [111], starvation-induced autophagy in *T. vaginalis* [112,113] and *T. cruzi* [114], glycosome turnover and damaged organelle removal by autophagy in *Leishmania* [115], and autophagy triggered by endoplasmic reticulum (ER) stress in *T. gondii* [116].

Notably, it was demonstrated that in apicomplexan parasites *P. falciparum* and *T. gondii* ATG8 takes on an autophagy-independent role associated with the apicoplast. The apicoplast is a non-photosynthetic plastid that is characteristic of most apicomplexans and is a product of secondary endosymbiosis between an apicomplexan ancestor and a chloroplast-containing red alga. Following significant reduction, the apicoplast emerged as the stroma and circular genome of the red alga endosymbiont’s plastid surrounded by four membranes. Although the role of the apicoplast appears to diminish in a number of apicomplexans, resulting in its devolution, this organelle remains essential for survival of the parasites with complex life cycles that involve switching between different hosts and tissue types, such as *Plasmodium* and *Toxoplasma* [117,118]. 

It was observed that ATG8 localises to the apicoplast, in all likelihood as the ATG8-PE conjugate, in *P. falciparum* [119,120,121] and *T. gondii* [122,123]. Upon *Tg*ATG8 depletion, *T. gondii* displayed growth cessation and defective segregation of the apicoplast into progeny, leading to loss of the organelle and prompting a link between this Ubl and apicoplast maintenance [122]. The crucial role of *Pf*ATG8 was demonstrated by the lethality of *Pf*ATG8 knockout to blood stage *P. falciparum*. The ensuing parasite death is due to apicoplast loss, likely brought on by inability to segregate the apicoplast during replication [124]. It was also reported that *Pf*ATG7 depletion inhibits parasite proliferation [124,125], and *Tg*ATG4 [123] is required for parasite growth and apicoplast maintenance, further supporting the essential role of the ATG8 pathway towards ensuring the proper function of this unusual organelle. The link between the ATG8 pathway and crucial parasite-specific process of apicoplast biogenesis has opened new avenues in anti-apicomplexan drug design. Upon structural analysis of the interaction between *Pf*ATG8 and its cognate E2 *Pf*ATG3 in *P. falciparum*, the structural loop that mediates the interaction was discovered in *Pf*ATG8, but not in its human counterpart [126]. This distinguishing feature enabled the development of parasite-specific inhibitors that selectively block *Pf*ATG8-*Pf*ATG3 interaction in *P. falciparum* and inhibit parasite growth [126,127,128,129].

Strikingly, the ATG12 pathway in apicomplexan parasites exhibits a significant divergence from the conventional one. The absence of ATG10 and the C-terminal glycine of ATG12 in these parasites pointed to the lack of functional ability to perform covalent attachment of ATG12 to ATG5. Instead, it was demonstrated that ATG12 and ATG5 orthologs from *P. falciparum* and *T. gondii* form a complex via non-covalent interaction, while retaining the ability to mediate ATG8 lipidation. This alteration in the ATG12 pathway in apicomplexans may be an example of reductive evolution leading to simplification of an energy-demanding Ubl pathway with a very limited substrate range to a non-covalent interaction [130].

## 5. URM1: The Old-Timer

URM1 is the ancestral Ubl that is most closely related to MoaD and ThiS, the prokaryotic antecedents of ubiquitin and Ubls. Through a mechanism reminiscent of the activity of these bacterial sulfur carriers, URM1 thiolates some eukaryotic tRNAs, thereby modulating their stability and functional properties [18,131]. Furthermore, URM1 functions as a protein PTM by modifying lysine residues of its substrates [18,131], mediated by a single E1-like enzyme Uba4 containing an additional rhodanese-like domain that confers sulfurtransferase activity [132]. Strikingly, no URM1-specific E2, E3, or deURMylase enzymes have been identified so far. It was discovered that URMylation is significantly induced by oxidative stress and targets a limited set of specific protein substrates. Based on its structural and functional features, URM1 may be considered an evolutionary intermediate that combines the roles of prokaryotic sulfur carriers with those of eukaryotic post-translational modifiers [18,131].

The NMR structure of *Trypanosoma brucei* URM1 revealed a conserved MoaD/ThiS-related fold and the close structural similarity to bacterial MoaD and *S. cerevisiae* URM1 [133]. Furthermore, URM1 and Uba4 have been bioinformatically predicted and functionally characterised in *Leishmania donovani* [134]. *L. donovani* that expressed a non-conjugatable form of *Ld*URM1 presented with reduced growth and defective cell division, indicating that this Ubl may be involved in the regulation of cell division. Twenty six proteins that may represent URMylation substrates in this organism were identified by mass spectrometry following immunoprecipitation with an anti-*Ld*URM1 antibody. The presence of *Ld*Rab5 and *Ld*Rab GTPase among these proteins, as well as the enrichment of *Ld*URM1 in proximity of the flagellar pocket, prompted a speculation that *Ld*URM1 may modulate endosome-mediated haemoglobin uptake through a specific receptor located in the flagellar pocket of *L. donovani* [134]. Although an URM1 ortholog exists in all other parasitic protozoans, its function has not been explored in these organisms to date.

## 6. UFM1: Expect the Unexpected

A relatively new addition to the Ubl family, UFM1, is conserved in multicellular organisms, but is absent in yeast. The attachment of UFM1 to lysines of its protein substrates is mediated by the E1 enzyme Uba5, the E2 enzyme Ufc1, and the E3 enzyme Ufl1. The UFM1-specific proteases, Ufsp1 and Ufsp2, function both as UFM precursor processing enzymes and deUFMylases. Notably, the UFM1 pathway is closely linked to ER function, as demonstrated by ER localisation of the transmembrane E3 Ufl1 and the upregulation of the pathway components by ER stress. While the pool of reported UFM1-modified proteins is small, this Ubl was shown to be crucial for the ER stress response, cell development and differentiation, and tissue homeostasis [135,136].

Given its association with multicellular organisms, the discovery of a UFM1 ortholog, as well as active UFMylation enzymatic machinery, in *Leishmania donovani* [137,138] was surprising. Unexpectedly, it was found that *Ld*UFM1 and its cognate enzymes are associated with the mitochondria, as evidenced by their cellular localisation and the detection of two mitochondrial proteins, the 40S ribosomal protein SA and the mitochondrial trifunctional protein (MTP) α-subunit, in *L. donovani* lysate fractions enriched for *Ld*UFM1 conjugates [137]. *Ld*MTP was subsequently confirmed as a genuine in vivo UFMylation substrate in this parasite [139]. The role of *Ld*UFM1 in the mitochondrial processes was further underscored by the disruption of the essential MTP-mediated β-oxidation of fatty acids in *L. donovani* UFM1 null mutant strain (Ufm1-/-). Lack of *Ld*UFM1 in this strain led to the inhibition of *L. donovani* amastigote growth in vitro and ex vivo in human macrophages, suggesting that *Ld*UFM1 is required for cell division and pathogenesis [139]. Furthermore, the *L. donovani* strain that lacks Ufsp, the *Ld*UFM1-specific protease, displayed similarly compromised growth in vitro and ex vivo, as well as reduced virulence in mouse infections [138]. This finding suggests that the Ufsp knockout strain of *L. donovani* is an attractive candidate to be tested as a live attenuated vaccine. Moreover, it was demonstrated that the activity of *Ld*Ufsp can be inhibited by the anti-leishmanial drug amphotericin B, thereby supporting the validity of this enzyme as a chemotherapy target [138]. UFM1 has now been bioinformatically predicted to function in *Trypamosoma*, *Giardia*, and *Toxoplasma*, although its biological function has not been studied in these organisms to date.

## 7. PUBL: The New Kid on the Block?

An extreme example of Ubl divergence in protozoans is plastid ubiquitin-like protein (PUBL), a novel, apicoplast-targeted modifier with high homology to ubiquitin that was discovered in *Toxoplasma gondii* [140]. Putative homologs of PUBL were identified in multiple apicomplexan parasites, with the exception of *Plasmodium*. It was demonstrated that PUBL is essential for *T. gondii* growth and protein import into the apicoplast, pointing to a potential link with the apicoplast-specific endoplasmic reticulum-associated protein degradation (ERAD) complex, known to mediate import into this organelle [140]. Unlike the conventional ERAD systems that function as the ER quality control mechanisms of the secretory pathway and target misfolded proteins for degradation, the algal endosymbiont-derived apicoplast ERAD complex is repurposed for protein import into the organelle. In addition to its divergence, the functional apicoplast ERAD system is essential for *T. gondii* survival [141], and PUBL conjugation machinery may thus hold potential as novel targets for anti-apicomplexan drug development.

## 8. Conclusions and Future Perspectives

**Figure 2:**
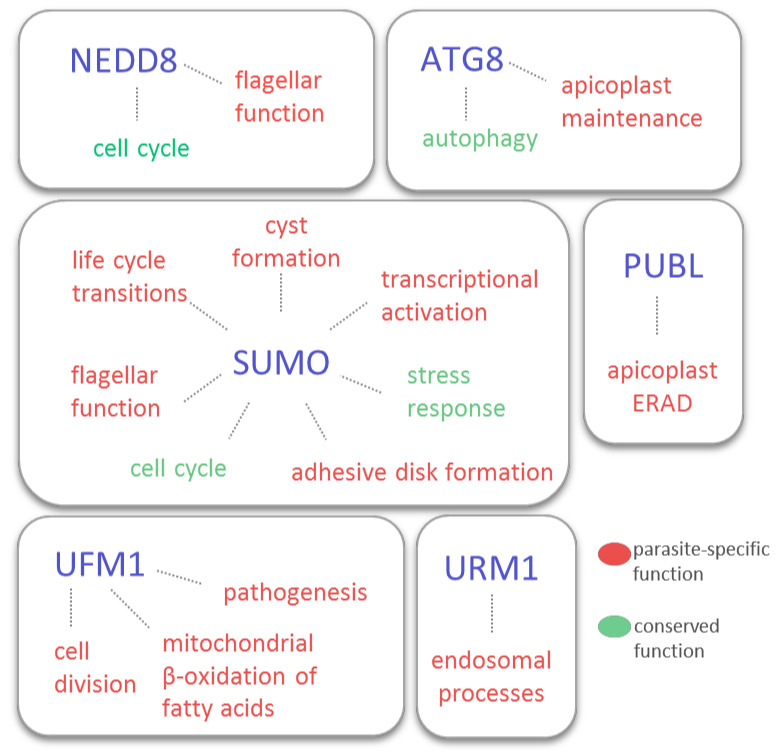
Cellular processes regulated by ubiquitin-like modifiers (Ubls) in protozoan parasites.

Parasitic protozoa employ a variety of Ubls that are structurally conserved across eukaryotes as post-translational modifiers. SUMO, NEDD8, and URM1 have been identified in all major lineages of these parasites, while ATG8, ATG12, and UFM1 are not uniformly present. For some Ubls, functional conservation is observed in mammalian cells and model organisms, as well as in protozoan parasites, as exemplified by SUMO and ATG8 that regulate stress responses and autophagy, respectively. However, these highly specialised unicellular parasites also utilise Ubls in unorthodox ways to facilitate unique processes that underpin their unusual physiology (Figure 2). The diversity of novel regulatory functions performed by Ubls reflects the diversity of cellular organisations and lifestyles in this polyphyletic group of parasites. Remarkably, multiple Ubls control the formation and function of parasite-specific organelles and life forms. In particular, SUMO mediates adhesive disc formation and encystation in *G. lamblia*, ATG8 is essential for apicoplast maintenance in apicomplexan parasites, while both NEDD8 and SUMO are required for normal flagellar function in trypanosomes. Furthermore, some Ubls in parasitic protozoa perform unexpected functions that are not related, or even contrary, to the roles of their counterparts in other organisms. Chromatin SUMOylation is linked to transcriptional activation of *VSG* in *T. brucei*, even though in other organisms this modification mainly leads to transcriptional repression. UFM1 regulates mitochondrial processes in *L. donovani*, in contrast to its conventional role in ER function. In addition to the functional divergence of Ubls, parasitic protozoa demonstrate distinctive features in Ubl enzymatic pathways, such as the replacement of ATG12 conjugation to its ATG5 substrate with a non-covalent interaction, and lack of CSN in apicomplexans.

Since Ubls are essential in facilitating a range of parasite-specific processes, enzymes mediating the Ubl pathways may hold therapeutic potential as targets for development of drugs against diseases caused by parasitic protozoa. Notably, Ubls have been implicated in the mode of action of multiple existing drugs that show anti-parasite activity, including benzoxaborole AN13762, monensin, and the isocryptolepine derivative ICL-M. Furthermore, the presence of distinctive structural features on the interacting surfaces of cognate enzymes allows specific targeting of parasitic Ubl pathways, while leaving host Ubl pathways unaffected. Such selective targeting is very promising from a drug design perspective and has been achieved for SUMO and ATG8 pathways through inhibition of Uba2–Uba9 and ATG8–ATG3 interactions in *P. falciparum*. Furthermore, given the evolutionary conservation and necessity of these pathways to survival in all eukaryotes, it would follow that these Ubls are essential across the entire life cycle of parasitic protozoans. As such, developing therapeutics that target specific components of Ubl machinery may yield inhibitors that prove effective against multiple stages of parasite development, something that has been a challenge with currently available drugs. 

From the data on Ubls in parasitic protozoa that have accumulated to date, we can glean the significance of these underexplored PTMs for the cellular physiology of parasites and potential applicability of Ubl pathways for drug development. Although these pathways are evolutionarily conserved, it is becoming increasingly evident that Ubls regulate distinct cellular processes in different species of parasitic protozoa and that their cognate enzymatic machinery displays unique features. This functional and structural divergence necessitates further research into these modifiers in a variety of parasitic protozoa, with the aim of improving our understanding of the regulatory functions of Ubls across the whole spectrum of these pathogenic organisms and harnessing their essential and unique roles for novel drug design.

## Figures and Tables

**Table 1:** The range of Ubls identified in different protozoan parasites. The presence or absence of each Ubl is denoted by ‘+’ or ‘−’, respectively.

	SUMO	NEDD8	ATG8	ATG12	URM1	UFM1	ISG15	FAT10
*Plasmodium*	+	+	+	+	+	−	−	−
*Toxoplasma*	+	+	+	+	+	+	−	−
*Trypanosoma*	+	+	+	+	+	+	−	−
*Leishmania*	+	+	+	+	+	+	−	−
*Giardia*	+	+	−	−	+	+	−	−
*Entamoeba*	+	+	+	−	+	−	−	−
